# Nasal Blockage and Academic Performance Among Medical College Students in the Kingdom of Saudi Arabia

**DOI:** 10.7759/cureus.36135

**Published:** 2023-03-14

**Authors:** Ali A Alshehri, Faisal Hakami, Walaa H Algadhi, Hussam Darraj, Sulaiman Hamdi, Mohammed Awaf, Alshomokh Hakami, Basem Zogel

**Affiliations:** 1 Surgery, College of Medicine, Najran University, Najran, SAU; 2 Surgery, College of Medicine, Jazan University, Jazan, SAU; 3 Medicine and Surgery, Jazan University, Jazan, SAU

**Keywords:** medical students, academic, otolaryngology, nasal blockage, obstruction sleep apnea

## Abstract

Objectives

The most frequent otolaryngological complaint is nasal obstruction. We aimed to determine if there is a relationship between nasal blockage and academic performance among medical college students in Saudi Arabia.

Methods

This cross-sectional survey carried out from August to December 2022, included 860 medical students determining the risk of obstructive sleep apnea (OSA) on the participants using the Berlin Sleep Questionnaire Risk Probability, then comparing it to their socio-demographic characteristics, while the Chi-square test was used for the comparison of categorical variables.

Result

The average age of the participants in our study was 21.52 years; 60% were females and 40% were males. The risk of obstructive sleep apnea was found to be two times higher in females than in males (95% CI: 1.195- 3.345; p-value 0.008). The hypertensive participants had a 27-fold increased risk of OSA compared to non-hypertensives. Grade Point Average (GPA) and snoring had a statistically significant relationship, however, a fifth of the participants reported snoring (79.8% reported not snoring). We also found that 14.8% of the participants with snoring had a GPA between 2-4.49 compared to 44.6% of participants without snoring.

Conclusion

Female students had a two-fold higher chance of developing OSA than males. While a GPA of 4.5 and above was more often associated with participants without snoring, the number of individuals with a GPA of 2-4.49 was greater among participants with snoring. To aid in the prevention of illness complications and the management of risk factors, additional efforts should be made to increase disease knowledge among students, primary healthcare practitioners, and specialty doctors.

## Introduction

Nasal blockage is the most common otolaryngological complaint, affecting about one-third of the population [1,2]. There are many factors that cause it, including anatomic and physiologic factors. The anatomic and physiologic factors affect the nasal passages, airflow receptors, mucociliary and autonomic function.

The anatomic causes include septal deviation or lesions in nasal passages such as enlarged adenoids or nasal polyps, internal valve stenosis, external nasal valve collapse, and turbinate hypertrophy [1-3]. The prevalence of nasal valve collapse, septal deviation, and inferior turbinate hypertrophy was about 67%, 76%, and 72%, respectively [4]. Chronic abnormalities such as septal deviation and nasal valve obstruction have resulted in sleep apnea [5]. Furthermore, obstructive sleep apnea (OSA) is aggravated by nasal obstruction by preventing airflow [6]. Obstructive sleep apnea is a common disorder characterized by nocturnal breathing cessation caused by upper airway collapse [7].

The most common complaints are snoring, sleep disruption, and excessive daytime sleepiness. Throughout the night, cycles of sleep, snoring, obstruction, arousal, and sleep occur. Some patients with severe apnea may have 100 or more episodes of upper airway obstruction in one hour [8].

Excessive daytime sleepiness in patients with OSA is most likely caused by multiple arousals and sleep fragmentation. Obstructive sleep apnea may go undiagnosed because many patients are unaware of their snoring and nocturnal arousals [9]. The prevalence of OSA in the Western world ranges from 3% to 7% in men and 2% to 5% in women. Despite lower obesity rates, the prevalence is comparable in Asia, possibly due to craniofacial differences. This finding suggests that OSA is a disorder that affects people in both the developing and developed worlds [10]. Nonetheless, few Middle Eastern studies have estimated the prevalence of sleep apnea. Obesity and sleep-related breathing disorders are on the rise in the Middle East including UAE, Kingdom of Saudi Arabia (KSA), Qatar, Bahrain, Kuwait, and Oman, with sedentary/Westernized lifestyles, fast food (high in fat, salt, sugar, and refined starches), adverse outdoor weather conditions, genetics, and a complex interplay of many other factors all playing a role [11].

Male gender, being overweight, having a narrow airway, drinking alcohol, nasal problems, and a family history of snoring or OSA are the most important risk factors [12]. In a sample of Saudi middle-aged adults, the prevalence and risks of habitual snoring and obstructive sleep apnea symptoms were present in 18.2% of females and 81.8% of males. 40% of the 2682 subjects enrolled snored: 23.5% were habitual snorers, 16.6% were moderate snorers, and 59.9% were non-snorers [13,14]. Sleep is essential for children and adolescents' learning, memory processes, and academic performance [15]. According to studies, poor sleep quality, sleeping late, getting up early, and interrupted nights affect behavior, learning capacity, and school performance [16].

A Jordanian study, published in 2018, was conducted among students at the Jordan University of Science and Technology to estimate the prevalence of symptoms and risk of OSA in association with poor academic performance. The study found that male students were more likely to suffer from OSA and more likely to have snoring compared to female students. These findings were consistent with poor academic performance [7]. A cross-sectional study was conducted between May and September 2019 at Al Qaboos University in Oman among 637 students aged between 17-25 years, and the prevalence of OSA was evaluated. The study found that those with OSA had low academic performance [17].

A cross-sectional study was conducted to estimate the prevalence of sleep among medical students at Umm Al-Qura University in KSA, including OSA, on 438 medical students from the second year to the sixth year of the study. The study found that OSA is common among medical students and negatively affects academic performance [18]. A narrative review aimed at shedding light on the impact of OSA on society, which also includes twice the academic average of university students, was concluded. Data were collected from relevant articles in the following databases: MEDLINE, Google Scholar, Scopus, SAGE Research Methods, and ScienceDirect. Data were also collected from official national reports and relevant websites such as the Federal Motor Carrier Safety Administration and the American Academy of Sleep Medicine (AASM). Analyzing 16 articles from 12 countries, the authors found that obstructive sleep leads to poor academic performance in core academic areas [19]. This study aimed to determine if there is a relationship between nasal blockage and academic performance among all medical college students all over the Kingdom of Saudi Arabia.

## Materials and methods

Study design and participants

This is a cross-sectional study conducted on medical science students in Saudi Arabia during the academic years 2022 and 2023. In this study, to identify OSA symptoms and calculate the prevalence of OSA, the Berlin Sleep Questionnaire (BSQ) was used, where a web-based Arabic language self-administered questionnaire was used to collect data. We adopted and modified the questionnaire, which requires their agreement, to make it suitable for our participants. The questionnaire's link was distributed to the target audience to determine the association between nasal blockage and academic performance. The information gathered was anonymous, and neither personally identifiable information nor sensitive information was collected. The questionnaire was divided into three sections: 1) the socio-demographic characteristics of participants, 2) the risk of developing obstructive sleep apnea, and 3) the impact of nasal blockage on academic performance, each of which has questions that were aimed to assess a single item. A pilot sample (n = 20) was used to clarify the length, amount of time, and ability required to complete the survey, and catch grammatical errors in the questionnaire items before the survey was actually distributed. It is notable that the study's analysis did not include the data from this pilot group.

Ethical approval

Ethical approval for conducting this study was obtained from the Najran Scientific Research Ethical Committee (reference number; 444-42-5438-DS, dated 26 August 2022). Consent was taken from all participants prior to their participation in the study.

Sample size and statistical analysis

The sample size was optimized using the Raosoft sample size calculator (Raosoft Inc., Seattle, USA) and using a convenience sample collection method, where we randomly collected 860 responses from medical colleges in the Kingdom of Saudi Arabia. All medical science students all over the Kingdom of Saudi Arabia were included in this study. Students from other subject areas were excluded from this study. The findings were analyzed using the SPSS for Windows version 23 (IBM Corp., Armonk, USA). The analysis was performed with a 95% confidence interval, and statistical significance was set at p<0.05. Data were presented as frequencies and percentages for categorical variables. Continuous variables were presented as mean ± SD, minimum and maximum values. Binary Logistic Regression was used for the adjusted analysis. Their risk of obstructive sleep apnea was determined using the Berlin Sleep Questionnaire Risk Probability, then compared to their socio-demographic characteristics. The Chi-square test was used for the comparison of categorical variables. We determined the socio-demographic data in the form of age, gender, nationality, place of residency, college, university level, BMI, and whether the participants are smokers, have chronic diseases, and family history of nasal blockage.

## Results

Socio-demographic characteristics of our study participants

A total of 860 participants were included in our study data analysis. The mean age of our study participants was 21.52±2.17 years with 60% and 40% females and males, respectively. Almost all of our study participants were Saudi and half of them were living in the southern region of the Kingdom of Saudi Arabia. One-third of our study subjects were students in the faculty of medicine. Regarding their medical issues, almost half of our study participants’ BMI was in the normal category, 740 (86%) were not smokers, and 176 (20%) reported a chronic medical illness (hypertension was the highest reported by 7.9%). In our study subjects, the participants who reported a family history of nasal blockage were only 7.7% (Table 1).

**Table 1:** The socio-demographic characteristics of our study subjects

Socio-demographic characteristics	n = 860	%
Age; mean ±S.D		
21.52±2.167		
Gender		
Male	332	61.4%
Female	528	38.4%
Nationality		
Saudi	838	97.4%
Non-Saudi	22	2.6%
Place of Residency		
The central region	149	17.3%
The western region	219	25.5%
The north region	64	7.4%
The eastern region	29	3.4%
The south region	399	46.4%
College		
College of medicine	285	33.1%
College of density	52	6%
College of pharmacy	143	16.6%
College of nurse	141	16.4%
College of applied medical sciences	203	23.6%
College of public health and informatics	36	4.2%
University Level		
First-year	143	16.6%
Second year	138	16%
Third year	129	15%
Fourth year	183	21.3%
Fifth year	110	12.8%
Sixth year	63	7.3%
Intern	94	10.9%
BMI		
Normal	479	55.8%
Underweight	130	15.1%
Overweight	176	20.5%
Obese	74	8.6
Smoking		
Yes	120	14%
No	740	86%
Chronic Diseases		
Hypertension	68	7.9%
Diabetes	32	3.7%
Respiratory Diseases	60	7%
Cardiac Diseases	12	1.4%
Thyroid Diseases	1	0.1%
Anemia	3	0.3%
Family History of Nasal Blockage		
Yes	66	7.7%
No	794	92.3%

The risk of developing obstructive sleep apnea among our study subjects

Binary Logistic Regression was used for the adjusted analysis and determining the risk of OSA using the Berlin Sleep Questionnaire Risk Probability, then compared to their socio-demographic characteristics as shown in Table 2. We found females were two times higher in risk of OSA compared to males (95% CI: 1.195- 3.345; p=0.008). The adjusted OR showed that participants with hypertension were associated with more risk of OSA by 27 times (95% CI: 15.261-50.299; p<0.001) compared to those with no hypertension. More importantly, the regression demonstrated that participants with snoring were at 40 times higher risk (95% CI: 20.419-78.528; p<0.001), and that participants who are suffering from a sudden interruption of their breathing process during sleep revealed three times higher risk (95% CI: 1.621-5.148; p<0.001).

**Table 2:** Binary logistic regression used to demonstrate the risk of developing obstructive sleep apnea among our participants by comparing the risk probability of the Berlin Sleep Questionnaire to the baseline characteristics of our study subjects *Alpha criterion was set to 0.05 or less to be considered a significant value aOR: adjusted odds ratio

The predictor variables	Risk Probability		aOR	CI (95%)	p-value*
	High Risk	Low Risk			
Gender					
Male	21	310	1		
Female	63	465	2	1.195- 3.345	0.008*
BMI					
Underweight	8	122	1		
Normal	47	432	1.659	0.764-3.605	0.201
Overweight	23	153	2.292	0.991-5.305	0.053
Obese	6	68	1.346	0.448-4.039	0.597
Diabetic mellitus					
No	76	751	1		
Yes	7	24	1.5	0.316-7.241	0.601
Hypertension					
No	33	640	1		
Yes	33	40	27.706	15.261-50.299	<0.001*
I don’t know	11	107	1.994	0.978-4.065	0.058
Family history of nasal blockage					
No	83	710	1		
Yes	1	65	0.067	0.009-0.521	0.010*
Smoking					
No	60	679	1		
Yes	24	96	1.427	0.604-3.373	0.417
Academic Grade					
Less than 2	2	12	5.400	0.760-38.356	0.092
2 to 2.74	4	21	5.060	0.824-31.085	0.080
2.75 to 3.74	25	121	2.953	1.211-7.205	0.017*
3.75 to 4.49	34	306	2.131	0.953-4.766	0.065
4.5 to 5	19	315	1		
In which situation do you think that your academic performance is affected					
During studying	49	282	0.607	0.207-1.781	0.363
During attendance of classes	28	76	1.871	0.894-3.916	0.097
Snoring					
No	11	674	1		
Yes	73	101	40.044	20.419-78.528	<0.001*
Did you notice that you stopped breathing during sleep, and couldn't back again to sleep?					
No	30	492	1		
Yes	48	200	2.889	1.621-5.148	<0.001*
I don’t know	6	83	1.070	0.382-2.994	0.898

The impact of nasal blockage on the academic performance of our study subjects

The Chi-square test showed that there is a significant association (p≤0.001) between grade point average (GPA) and snoring. We found that 20.2% of our study participants reported that they had snoring. Among those participants, we observed that 14.8% had a GPA between 2-4.49 compared to participants without snoring (79.8% of study participants), among which 44.6% had that GPA. In other words, as demonstrated in Table 3, a GPA of 2-4.49 was higher among participants with snoring, whereas a GPA of 4.5 and above was more associated with participants without snoring. Regarding the risk of obstructive sleep apnea and smoking, the prevalence rate of participants with high risk and smokers was 9.8% and 14%, respectively. The distribution of the participants with higher risk and smoking was more toward GPA less than 4.5, whereas participants with lower risk and non-smokers were distributed more toward a GPA of 4.5 or more, as shown in Table 3.

**Table 3:** Chi-square test was used to identify a significant association between nasal blockage and its major risk factors with academic performance GPA: grade point average *Alpha criterion was set to 0.05 or less to be considered a significant value

Variable	GPA					
	Less than 2 (n = 14)	from 2 -2.74 (n = 25)	from 2.75 - 3.74( n = 146)	from 3.75-4.49 (n = 340)	from (4.5-5 n = 335)	p-value*
Snoring						
Yes (n = 174, 100%)	1.1%	5.7%	24.7%	42.5%	25.9%	<0.001*
No (n = 686, 100%)	1.7%	2.2%	15%	38.8%	42.3%	
Risk of obstructive sleep apnea						
High Risk (n = 84 100%)	2.4%	4.8%	29.8%	40.5%	22.6%	0.001*
Low Risk (n = 775, 100%)	1.5%	2.7%	15.6%	39.5%	40.6%	
BMI						
Underweight (n = 130, 100%)	2.3%	2.3%	12.3%	16.8%	14.6%	0.9
Normal (n = 480, 100%)	1.3%	3.1%	56.8%	55.3%	56.1%	
Overweight (n = 176, 100%)	2.3%	2.8%	22.6%	18.8%	20.9%	
Obese (n = 74, 100%)	1.4%	2.7%	8.2%	9.1%	8.4%	
Smoking						
Yes (n = 120, 100%)	1.7%	7.5%	24.2%	44.2%	22.5%	<0.001*
No (n = 740, 100%)	1.6%	2.2%	15.8%	38.8%	41.6%	
Hypertension						
Yes (n = 68, 100%)	2.9%	1.5%	22.1%	39.7%	33.8%	0.3
No (n = 673, 100%)	1.5%	2.5%	15.8%	40.1%	40.1%	
I don’t know (n = 119, 100%)	1.7%	5.9%	21%	36.1%	35.3%	

## Discussion

Congestion and nasal block are considered common clinical conditions; however, patients and doctors define the term very differently. Congestion can be described as fullness, stuffiness, or obstruction of the nasal cavity. But a blockage usually means a blockage that cannot be repaired. Nasal congestion is not limited to allergic rhinitis; however, It is a common symptom and problematic in other conditions that affect the sinonasal passages, including rhinosinusitis [20]. The economic effects of nasal blockage and the specific burden on quality of life (QOL) and academic performance are not well studied. Therefore, the aim of the current study was to study the association between nasal blockage and academic performance among medical college students in the Kingdom of Saudi Arabia.

In the current study, the prevalence of snoring among students in medical colleges was 20.2%, while the prevalence of students with a high risk of obstructive sleep apnea was 9.78%. The risk factors of having a high risk of obstructive sleep apnea included being female (two times higher risk), hypertension (higher risk by 27 times), snoring (40 times higher risk), and sudden interruption of their breathing process during sleep (three times higher).

In a previous systematic review, the authors reported that risk factors associated with OSA included being more than 35 years old, having a BMI of more than 25 kg/m2, being alcoholic, having a higher Epworth Sleepiness Scale (ESS), having a higher mean apnea duration, having higher oxygen desaturation index (ODI), and having nocturnal oxygen desaturation (NOD) [21]. In addition, another study reported that the frequency of apnea in women with gestational hypertension was 1.4 times higher than the control group without hypertension [22], and in a community prevalence study, the authors showed that male gender, advancing age, obesity, and an increased waist-to-hip ratio are important risk factors for OSA [23].

In another cross-sectional study, the authors found that 20% of participants were classified as at high risk for OSA, with more males than females at high risk, while OSA was more common in the elderly, people who are obese, smoke, and spend more time watching TV, and have poor self-consciousness about physical health [24]. Previous systematic reviews, including studies that objectively measured OSA in adults in the general population using laboratory instruments, showed a wide variety of OSA prevalence, with estimates for moderate OSA ranging from 9% to 38%, and estimates for moderate to severe OSA ranging from 6% to 17% [25]. Moreover, in another study that used the Berlin Sleep Questionnaire on university students in Chile, the authors found that 7.8% of university students were at risk of developing OSA [26]. Focusing on published studies on Asian adults, the reported prevalence of OSA ranged from 3.7% to 97.3% [27].

A study of a large sample of school personnel in Saudi Arabia estimated the prevalence of OSA at 8.8% (men 12.8% and women 5.1%) based on a prevalence assessment [28]. This wide variation in the reported prevalence of OSA may be explained in part by verification methods. In addition, the results of the 2005 National Sleep Foundation Sleep of America survey found that 26% of participants met the criteria for a Berlin survey of significant risk of OSA [29]. A national survey conducted among the Korean adult population reported that 15.8% of participants were classified as having a high risk of OSA as defined by the Berlin Questionnaire [30]. 

In addition, another study conducted in a primary care setting in the United Arab Emirates showed that the prevalence of high risk of OSA was 20.9% [31]. However, comparisons of population-wide OSA prevalence estimates should be interpreted with caution due to the nature of the study subjects and the method of investigation, which may lead to marked differences between populations. Moreover, we found that there is a significant association (p≤0.001) between GPA and snoring where a GPA of 2-4.49 was higher among participants with snoring, whereas a GPA of 4.5 and above is more associated with participants without snoring. Furthermore, the distribution of participants with higher risk and smoking was more toward GPA less than 4.5, whereas participants with lower risk and non-smoking were distributed more towards GPA more than 4.5. These results showed that nasal block causing symptoms of snoring or high risk of obstructive apnea is associated with a significant negative impact on academic performance. In a previous study, the authors showed that poor control of nasal blockage and rhinitis was associated with a higher negative impact on the academic productivity of the students and a higher percentage of missed education hours because of the allergy [32].

The same results were reported by another study which showed a significant association between the loss of academic productivity and the severity of allergic rhinitis and nasal blockage [33]. In addition, another cross-sectional study conducted among 777 students showed that self-reported snoring and being at high risk for OSA were associated with poor academic performance [7]. Moreover, another study that aimed to study the impact of common sleep disorders on students’ academic performance showed that OSA was uncommon; however, one-third of the study's participants, who were at risk for obstructive sleep apnea, were at academic risk of having GPA of lower than 2.0 [34]. These results are similar to our study as well as other studies that showed that the presence of sleep-disordered breathing is associated with academic failure [35] and another study that showed improvement in academic scores in patients with OSA after having tonsillectomy or adenoidectomy [36].

Our study had some limitations, including depending on self-reported questionnaires in the diagnosis of the medical condition. Depending on clinical examination to diagnose nasal apnea should be considered in future studies. Moreover, self-reported questionnaires may be associated with personal bias.

## Conclusions

This study highlights the negative effects of sleep apnea on academic performance; however, academic performance is a complex process that can be affected by a range of psychosocial factors. This study found that snoring and the risk of OSA were not common among young college students. Women were associated with twice the risk of OSA, and both snoring and the risk of OSA were associated with decreased academic performance. Obstructive sleep apnea should be considered in students with unexplained poor academic performance. Further investigation should be carried out during life crises.
